# Anaesthetists’ knowledge and awareness of diathermy use in a department of anaesthesiology

**DOI:** 10.4102/jcmsa.v3i1.225

**Published:** 2025-09-24

**Authors:** Harrilene Apleni, Juan Scribante, Helen Perrie, Zainub Jooma

**Affiliations:** 1 Department of Anaesthesia, Faculty of Health Sciences, School of Clinical Medicine, University of the Witwatersrand, Johannesburg, South Africa; 2 Divisions: Paediatric Surgery, Department of Surgery, Faculty of Health Sciences, School of Clinical Medicine, University of the Witwatersrand, Johannesburg, South Africa

**Keywords:** knowledge, diathermy, anaesthetists, principles, risks, precautions

## Abstract

**Background:**

Anaesthetists are expected to have a basic understanding of diathermy use. The aim of this study is to evaluate anaesthetists’ knowledge and awareness of diathermy use in the Department of Anaesthesiology at the University of the Witwatersrand, Johannesburg, South Africa.

**Methods:**

A prospective, descriptive and contextual study was conducted utilising an anonymous self-administered questionnaire distributed to anaesthetists during academic meetings, and convenience sampling was used. A minimum sample size of 96 anaesthetists was estimated. Using the modified Angoff Method, a score of 62% was determined for adequate knowledge.

**Results:**

One hundred and one questionnaires met the criteria for analysis. The overall mean score obtained for knowledge was 44.7%; 47.7% for junior anaesthetists *versus* 42.7% for senior anaesthetists (*p* = 0.20). The total number of anaesthetists achieving an adequate score was 13 (12.9%). Of those, 10 (76.9%) were junior anaesthetists with a mean score of 71.0%, and 3 (23.1%) were senior anaesthetists with a mean score of 67.0% (*p* = 0.72). There was no significant difference in the knowledge between anaesthetists with Fellowship of the College of Anaesthesia Part 1 examinations and those without (*p* = 0.34). In a comparison of knowledge between junior and senior anaesthetists, junior anaesthetists scored significantly better in the category of precautions and appropriate use (*p* = 0.02).

**Conclusion:**

The University of the Witwatersrand (Wits) anaesthetists demonstrated poor overall knowledge of diathermy. While anaesthetists do not apply diathermy pads or use diathermy themselves, they are responsible for patients’ safety in the operating theatre and diathermy may interfere with anaesthetic equipment or patient devices.

**Contribution:**

This study investigated anaesthetists’ knowledge and awareness pertaining to diathermy use in the operating theatre. It has highlighted the need for ongoing education pertaining to diathermy safety for anaesthetists.

## Introduction

Electricity is a phenomenon that has been used for many years in the operating theatre, and its current use is an intrinsic characteristic of modern surgery.^1^ Diathermy or electrocautery utilises the principles of electricity and is defined as ‘the cutting and coagulation of body tissue with a high frequency current’.^1,2^ Historically, diathermy use in medicine dates to 1925 when Cushing first contemplated its use during a medical conference when one of his residents suggested that an electrosurgical machine be used on the brain.^1,3^ A collaborative effort between Cushing and Bovie at Harvard University refined electrocautery and its use during neurosurgical procedures to limit blood loss. A report published indicating their success helped to bring diathermy to the attention of the medical field and it therefore revolutionised surgery.^1,3^

Although most modern diathermy machines are safe, the electric fields they create may pose hazards to the patients and operating theatre staff. They can cause burn injuries, electrocution, operating theatre fires, arrhythmias and smoke inhalation.^1,4^ The incidence of diathermy-induced injuries reported is 1–5 cases per 1 000 operations. Between 50 and 100 cases of surgical fires occur every year in the United States, with diathermy being the primary cause of such fires.^1,2^

An analysis by Kressin^5^ of the American Society of Anaesthesia Closed Claims Project Database of 2004 revealed that the incidence of inadvertent operating theatre burns was 2%. Electrocautery accounted for 19% of intraoperative burns, including both diathermy-induced fires and electric grounding pad skin burns.^1^ Sixty-four per cent of burns caused by diathermy were facial burns, and these represented 21% of all burn claims.^1,5^

Inadvertent burning of a patient is traumatic and often catastrophic, not only for the affected patient but equally for the operating theatre team.^1^ Physical scars, emotional trauma and prolonged hospital stay are inevitable, depending on the extent of the burn. Doctor–patient relationships often become disrupted with legal consequences to the relevant practitioners following a diathermy injury.^6^

While anaesthetists do not apply diathermy pads or use diathermy themselves, they are responsible for the well-being of patients in the theatre.^1^ Patient devices such as cardiac pacemakers and internal cardiac defibrillators could pose potential hazards in the presence of diathermy.^1^ Ensuring patient and staff safety by minimising risk is crucial and knowledge is the key to patient safety.^1,7^ Anaesthetists are expected to have a basic understanding of diathermy and the principles of diathermy use, operating theatre fires and electrical safety, which are all included in the curriculum of ‘Fellowship of the College of Anaesthetists (FCA) Part 1’.^1,8^ The aim of this study is to evaluate anaesthetists’ knowledge and awareness of the uses of diathermy in the Department of Anaesthesiology at the Witwatersrand (Wits).

## Research methods and design

This was a prospective, contextual, descriptive study using an anonymous self-administered questionnaire.

The study population consisted of all anaesthetists working in the Department of Anaesthesiology at University of the Witwatersrand (Wits). In consultation with a biostatistician, a minimum sample of 96 anaesthetists was calculated assuming that 65% of anaesthetists in the Wits Department of Anaesthesiology would have adequate knowledge of diathermy with a 10% margin of error and 95% confidence.^1,9^

In this study, a junior anaesthetist was defined as a medical officer or registrar in the first, second or third year of training, and a senior anaesthetist was a registrar with 4 or more years of training, a consultant or a career medical officer (doctor with a Diploma in Anaesthesia and 10 or more years of experience).^1^ No questionnaires pertaining to the knowledge and appropriate use of diathermy among anaesthetists were identified in the literature. A draft questionnaire was compiled based on a review of the literature to ensure content validity. The draft questionnaire was reviewed by three specialist anaesthetists to ensure face and content validity, and their suggested changes were incorporated into the questionnaire.

The final questionnaire consisted of two sections: Section 1 included the demographic data of anaesthetists, and Section 2 consisted of 15 questions regarding the knowledge and awareness of diathermy. The questions in Section 2 were divided into different knowledge categories: firstly, the principles of diathermy consisting of three questions; secondly, hazards and complications with six questions and, thirdly, a category on precautions and appropriate use of diathermy with six questions (Online Appendix 1).^1^

The questionnaires were distributed at departmental academic meetings by the principal investigator (HA) together with a participant information sheet, and the departmental members were invited to participate after explanation of the study. Completion of the questionnaire implied consent. Anonymity was maintained as no personal information was requested on the questionnaire. All the completed questionnaires were folded and placed in a sealed box at the door of the meeting room. A convenience sampling method was used. The questionnaires were numbered to calculate a response rate. One author (H.A.) handed out the questionnaires to prevent participants from completing more than one questionnaire and was present during the completion of questionnaires to assist with queries and to prevent data contamination. To further prevent data contamination, questionnaires were distributed at non-consecutive departmental meetings. Unanswered questions were considered incorrect.^1^

Adequate knowledge was determined as 62% using the Modified Angoff Method based on assessment by five consultants in the department who were involved in teaching and examining basic sciences in the department.^1,10^ Consensus for the percentage of participants who would correctly answer each question was achieved within 10% from each consultant involved. The average mark was then calculated to determine adequate knowledge for the questionnaire. Blank questionnaires were excluded from the study. Using Microsoft Excel 2010, data were captured into the spreadsheets. The statistical programme STATA Version 15 (StataCorp, USA) was used to analyse data. Categorical variables were described using frequencies and percentages and compared using chi-squared tests. Continuous variables were described using means and standard deviations and compared using independent *t*-tests. A *p*-value of < 0.05 was considered statistically significant.

### Ethical considerations

Ethical clearance to conduct this study was obtained from the University of the Witwatersrand, Human Research and Ethics Committee (Medical) on 18 March 2019. The ethical clearance number is M190107.

## Results

A total of 110 questionnaires were distributed at departmental academic meetings, of which 101 (91.8%) were returned. There were 59 (58.4%) junior and 42 (41.6%) senior anaesthetists who participated. The demographics of anaesthetists are shown in Table 1.

**TABLE 1:** Demographics of anaesthetists (*N* = 101).

Variable	*n*	%
**Professional designation**		
Consultant	26	25.7
Career Medical Officer	5	5.0
Registrar	58	57.4
Medical Officer	12	11.9
**Experience in Anaesthesia (years)**		
< 1	8	7.9
1–5	56	55.4
6–10	25	24.8
11–15	7	6.9
16–20	2	2.0
> 20	3	3.0
**Registrar time (year)**		
First	15	25.9
Second	14	24.1
Third	18	31.0
Fourth	11	19.0
**FCA Part 1 examinations**		
Yes	80	79.2
No	21	20.8
**How long ago passed FCA1 Part 1 examinations (years)**		
Not applicable	23	22.8
< 1	15	14.8
1–5	48	47.5
6–10	11	10.9
> 10	4	4.0

Source: From Anaesthetists’ knowledge and awareness of diathermy used in a department of anaesthesiology: Research report [homepage on the Internet]. Wits WiredSpace Repository. Available from: https://wiredspace.wits.ac.za/items/a3be90ab-28ac-4f67-9664-86cfa5fdbb31

FCA, Fellowship of the College of Anaesthetists.

The overall mean score obtained for knowledge was 44.7% (S.D. 16). There were 13 (12.9%) anaesthetists who achieved an adequate knowledge score. Of the total anaesthetists, 10 (76.9%) were junior anaesthetists with a mean score of 71.0% and 3 (23.1%) were senior anaesthetists with a mean score of 67.0%; the difference was not statistically significant (*p* = 0.72). Of those who achieved an adequate knowledge score, 10 (76.9%) had passed the FCA Part 1 examinations ≤ 5 years ago. The number of correct responses to the questions is presented in Table 2.^1^

**TABLE 2:** The number of correct responses to the questions.

Question description	Anaesthetists with correct responses					
	Total (*N* = 101)		Junior (*n* = 59)		Senior (*n* = 42)	
	*n*	%	*n*	%	*n*	%
**Principles of diathermy**						
Range of alternating frequency	16	15.8	10	9.9	6	5.9
Type of diathermy	64	63.4	36	35.7	28	27.7
Amount of heat generated	65	64.4	41	40.6	24	23.8
**Hazards and complications**						
Fire triangle	95	94.1	56	55.5	39	38.6
Minimally invasive surgery complications	58	57.4	33	32.7	25	24.7
Diathermy waveform	29	28.7	17	16.8	12	11.9
Operating room fire with oxygen use	57	56.4	33	32.7	24	23.7
Airway fire with 100% oxygen	43	42.6	25	24.8	18	17.8
Peripheral skin burns	36	35.6	22	21.8	14	13.8
**Precautions and appropriate use**						
Ground plate contact areas and wires	16	15.8	10	9.9	6	5.9
Advantage of bipolar diathermy	51	50.5	30	29.7	21	20.8
Diathermy and cardiac pacemaker	39	38.6	29	28.7	10	9.9
Patient safety from electrocution	10	10.0	8	8.0	2	2.0
Patient safety from electrical shock	59	58.4	39	38.6	20	19.8
Placement of return electrode	40	39.6	22	21.8	18	17.8

Source: From Anaesthetists’ knowledge and awareness of diathermy use in a department of anaesthesiology: Research report [homepage on the Internet]. Wits WiredSpace Repository. Available from: https://wiredspace.wits.ac.za/items/a3be90ab-28ac-4f67-9664-86cfa5fdbb31

The knowledge scores of the anaesthetists and the comparison of knowledge between the junior and senior anaesthetists for the questionnaire as a whole and per knowledge category are presented in Table 3. The only category where there was a statistically significant difference between junior and senior anaesthetists was precautions and appropriate use (*p* = 0.02). There was no difference in knowledge for the questionnaire as a whole between anaesthetists with Part 1 examinations and those without (*p* = 0.34).^1^

**TABLE 3:** Comparison of knowledge in different categories between the junior and senior anaesthetists.

Category	Score		%		*p*
	Mean	S.D.	Mean	S.D.	
**Overall**	-	-	-	-	0.20
All anaesthetists	6.7	2.4	44.7	16.0	-
Junior anaesthetists	7.0	2.5	46.7	16.7	-
Senior anaesthetists	6.4	2.1	42.7	14.0	-
**Principles of diathermy (3 points)**	-	-	-	-	0.55
All anaesthetists	1.4	0.8	46.7	26.7	-
Junior anaesthetists	1.5	0.8	50.0	26.7	-
Senior anaesthetists	1.4	0.7	46.7	23.3	-
**Hazards and complications (6 points)**	-	-	-	-	0.87
All anaesthetists	3.2	1.4	53.3	23.3	-
Junior anaesthetists	3.2	1.5	53.3	25.0	-
Senior anaesthetists	3.1	1.2	51.7	20.0	-
**Precautions and appropriate use (6 points)**	-	-	-	-	0.02
All anaesthetists	2.1	1.1	35.0	18.3	-
Junior anaesthetists	2.4	1.1	40.0	18.3	-
Senior anaesthetists	1.8	1.1	30.0	18.3	-

Source: From Anaesthetists’ knowledge and awareness of diathermy use in a department of anaesthesiology: Research report [homepage on the Internet]. Wits WiredSpace Repository. Available from: https://wiredspace.wits.ac.za/items/a3be90ab-28ac-4f67-9664-86cfa5fdbb31

**FIGURE 1:**
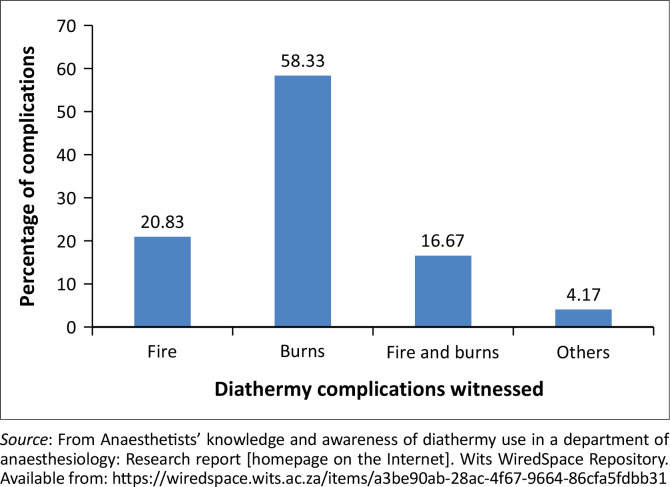
Complications witnessed during diathermy use.

Regarding awareness, more than 60% of the procedures that the anaesthetists were involved in required diathermy. Most anaesthetists, 88 (87.1%), responded that they thought diathermy was a safe instrument; however, 48 (47.8%) anaesthetists had witnessed complications. Figure 1 shows the complications witnessed during diathermy use. The complications under ‘other’ in Figure 1 were sparks as well as shock and burns to the surgeon’s hand.^1^

## Discussion

The results of this study indicate that the knowledge among anaesthetists in the categories: principles of diathermy, hazards and complications and precautions and appropriate use of diathermy was poor, with an overall mean score of 44.7%. Using a cut-off score of 62% for adequate knowledge, there were only 13 (12.9%) anaesthetists who achieved adequate knowledge. The poor scores were unexpected as diathermy use and principles form part of the FCA Part 1 curriculum^8^ and 65 (64.3%) anaesthetists indicated that they had passed this examination within the last 5 years.^1^

There was no difference between the overall knowledge of the senior and junior anaesthetists. The senior anaesthetists are role models for junior anaesthetists and oversee the teaching, and it would have been expected that they would be more knowledgeable regarding diathermy use because of their experience despite having written their FCA Part 1 examination many years ago.^1^

The least number of correct responses was for the question regarding patient safety from electrocution. Only 10% of anaesthetists answered this question correctly. Electrocution may result in cardiac arrhythmias, burns, nerve injury and minor complications such as stimulation of excitable tissues, lower limb movement during urological surgery or direct stimulation of the muscles causing contractions.^1,11^ Several measures can prevent or reduce the risk of electrocution, such as adequate maintenance and regular testing of diathermy, and ensuring the patient is not in contact with earthed objects, which is a role of anaesthetists in the operating theatre.^1,12^

Similarly, both the junior and senior anaesthetists answered poorly on how to use diathermy in a patient with a cardiac pacemaker and on identifying diathermy waveforms. Diathermy interferes with cardiac pacemakers and internal cardiac defibrillators by altering their electrical activity, which may result in ventricular fibrillation and other forms of arrhythmias.^1,13^ Furthermore, these devices may be permanently damaged by using diathermy.^14^ Failure to understand the effects of diathermy use poses a risk to patient safety, and adverse outcomes may result from incorrect use.

Diathermy is a commonly used instrument in the operating theatre, with the anaesthetists in this study indicating that it was required in more than 60% of the procedures they were involved in.^1^ Most of the diathermy-related adverse events are considered preventable by ensuring an understanding of the technology and the application and by being aware of the potential risks.^1,15^ Many complications are based on the faulty use of diathermy and its settings; knowledge and basic skills in operating these devices are of great importance.^1,16^

Studies examining the knowledge and awareness of diathermy among anaesthetists could not be identified. Several studies conducted among surgeons indicated poor knowledge regarding diathermy use irrespective of speciality, seniority or training.^1,2,15,17–20^ Mayooran et al.^19^ found that retention of knowledge after training was poor as the respondents demonstrated inadequate knowledge of diathermy a year later.^1^ Similarly, Pandey et al.^20^ surveyed obstetrics and gynaecology trainees regarding knowledge of diathermy and found that trainees had poor knowledge and that those who had attended a surgical skills course were not more knowledgeable.^1^ Assiotis et al.^17^ assessed diathermy training of surgical trainees and concluded that most of them had received no training on the basic principles and appropriate use of diathermy.^1^ Like our study, seniority played no role in better knowledge regarding diathermy use.^1,17^

The surgical smoke generated from diathermy can harbour chemicals including phenols, fatty acids and hydrocarbons as well as biological particles such as bacteria and viruses. There is sparse research on the actual levels of these particles in surgical smoke and the actual risk posed to theatre personnel is unknown. Awareness of this risk became more recognised during the coronavirus disease 2019 (COVID-19) pandemic.^21^ This concept was not interrogated in our questionnaire.

The contextual nature of this study is a potential limitation, as the study was conducted within the Department of Anaesthesiology at Wits and may not be generalisable to other departments in South Africa or other countries. While the questionnaire was designed based on existing literature and principles on diathermy relevant to the FCA Part 1 examination, content validity and reliability testing using the Cronbach’s alpha test were not performed. Comparisons were made between the junior and senior anaesthetists as defined in this study, rather than between trainees and specialists, as the specialist group was too small for meaningful analysis. This may limit the comparisons of the findings with other published literature. Furthermore, the study was powered for its adequacy of knowledge and not for comparative analysis, and therefore these results should be considered exploratory.

The authors recommend that the use of diathermy receive greater attention in the training programme of anaesthetic registrars and that it be part of continuous learning initiatives for anaesthetists.

## Conclusion

The Wits anaesthetists showed poor overall knowledge regarding the principles, hazards, complications, precautions and appropriate use of diathermy. While anaesthetists do not apply diathermy pads or use diathermy themselves, they are responsible for the safety of patients in the operating theatre, and therefore, diathermy may interfere with anaesthetic equipment or patient devices.
